# Tumor fraction-guided cell-free DNA profiling in metastatic solid tumor patients

**DOI:** 10.1186/s13073-021-00898-8

**Published:** 2021-05-31

**Authors:** Dana W. Y. Tsui, Michael L. Cheng, Maha Shady, Julie L. Yang, Dennis Stephens, Helen Won, Preethi Srinivasan, Kety Huberman, Fanli Meng, Xiaohong Jing, Juber Patel, Maysun Hasan, Ian Johnson, Erika Gedvilaite, Brian Houck-Loomis, Nicholas D. Socci, S. Duygu Selcuklu, Venkatraman E. Seshan, Hongxin Zhang, Debyani Chakravarty, Ahmet Zehir, Ryma Benayed, Maria Arcila, Marc Ladanyi, Samuel A. Funt, Darren R. Feldman, Bob T. Li, Pedram Razavi, Jonathan Rosenberg, Dean Bajorin, Gopa Iyer, Wassim Abida, Howard I. Scher, Dana Rathkopf, Agnes Viale, Michael F. Berger, David B. Solit

**Affiliations:** 1 Marie-Josée and Henry R. Kravis Center for Molecular Oncology, Memorial Sloan Kettering Cancer Center, 1275 York Avenue, New York, NY 10065 USA; 2 Department of Pathology, Memorial Sloan Kettering Cancer Center, 1275 York Avenue, New York, NY 10065 USA; 3 Weill Cornell Medical College, Weill Cornell University, New York, USA; 4 Present Address: PetDx, Inc., La Jolla, USA; 5 Department of Medicine, Memorial Sloan Kettering Cancer Center, New York, USA; 6 Present Address: Dana-Farber Cancer Institute, Harvard Medical School, Boston, USA; 7 Present Address: Graduate School of Arts and Sciences, Harvard University, Cambridge, USA; 8 Present Address: NYU Langone Health, New York, USA; 9 Department of Epidemiology-Biostatistics, Memorial Sloan Kettering Cancer Center, New York, USA; 10 Human Oncology and Pathogenesis Program, Memorial Sloan Kettering Cancer Center, New York, USA

**Keywords:** Liquid biopsy, Plasma DNA, Molecular diagnostic, Cancer, Sequencing, Noninvasive

## Abstract

**Background:**

Cell-free DNA (cfDNA) profiling is increasingly used to guide cancer care, yet mutations are not always identified. The ability to detect somatic mutations in plasma depends on both assay sensitivity and the fraction of circulating DNA in plasma that is tumor-derived (i.e., cfDNA tumor fraction). We hypothesized that cfDNA tumor fraction could inform the interpretation of negative cfDNA results and guide the choice of subsequent assays of greater genomic breadth or depth.

**Methods:**

Plasma samples collected from 118 metastatic cancer patients were analyzed with cf-IMPACT, a modified version of the FDA-authorized MSK-IMPACT tumor test that can detect genomic alterations in 410 cancer-associated genes. Shallow whole genome sequencing (sWGS) was also performed in the same samples to estimate cfDNA tumor fraction based on genome-wide copy number alterations using *z*-score statistics. Plasma samples with no somatic alterations detected by cf-IMPACT were triaged based on sWGS-estimated tumor fraction for analysis with either a less comprehensive but more sensitive assay (MSK-ACCESS) or broader whole exome sequencing (WES).

**Results:**

cfDNA profiling using cf-IMPACT identified somatic mutations in 55/76 (72%) patients for whom MSK-IMPACT tumor profiling data were available. A significantly higher concordance of mutational profiles and tumor mutational burden (TMB) was observed between plasma and tumor profiling for plasma samples with a high tumor fraction (*z*-score≥5). In the 42 patients from whom tumor data was not available, cf-IMPACT identified mutations in 16/42 (38%). In total, cf-IMPACT analysis of plasma revealed mutations in 71/118 (60%) patients, with clinically actionable alterations identified in 30 (25%), including therapeutic targets of FDA-approved drugs. Of the 47 samples without alterations detected and low tumor fraction (*z*-score<5), 29 had sufficient material to be re-analyzed using a less comprehensive but more sensitive assay, MSK-ACCESS, which revealed somatic mutations in 14/29 (48%). Conversely, 5 patients without alterations detected by cf-IMPACT and with high tumor fraction (*z*-score≥5) were analyzed by WES, which identified mutational signatures and alterations in potential oncogenic drivers not covered by the cf-IMPACT panel. Overall, we identified mutations in 90/118 (76%) patients in the entire cohort using the three complementary plasma profiling approaches.

**Conclusions:**

cfDNA tumor fraction can inform the interpretation of negative cfDNA results and guide the selection of subsequent sequencing platforms that are most likely to identify clinically-relevant genomic alterations.

**Supplementary Information:**

The online version contains supplementary material available at 10.1186/s13073-021-00898-8.

## Background

Molecular profiling of tumors using next-generation sequencing (NGS) is increasingly used to aid in diagnosis, guide treatment selection, and monitor disease status in patients with cancer. However, biopsies of primary or metastatic lesions may not be of sufficient quality for genomic analysis, or may fail to capture spatial and/or biologic heterogeneity or treatment-associated clonal evolution. Profiling of plasma cell free DNA (cfDNA) in body fluids can overcome many of these limitations [1, 2] by allowing for serial, minimally invasive sampling which can be used to identify targetable genomic alterations, monitor treatment response [3], detect minimal residual disease [4], or screen for cancer in high-risk populations [5, 6].

Negative cfDNA results must, however, be interpreted with caution. In patients with cancer, the cfDNA in plasma is derived from both tumor and normal cells, in particular white blood cells [7]. cfDNA tumor fraction, defined as the fractional proportion of tumor DNA relative to total cfDNA, is dependent on multiple factors, including disease extent (localized vs metastatic) [8], overall tumor burden, disease activity (progressing, stable or responding to systemic therapy) [9], patient-context factors such as fasting status or physical activity prior to blood collection [10], and technical pre-analytic factors related to sample acquisition, transport, and sample processing procedures [11], among others.

The likelihood of detecting a tumor mutation in plasma is dependent on (i) the cfDNA tumor fraction, (ii) the breadth and depth of the cfDNA assay employed, and (iii) the total number of tumor-derived mutations interrogated. Given the generally low fraction of tumor-derived DNA in plasma, many commercial cfDNA assays are designed to screen for a small number of actionable genomic alterations through ultra-deep sequencing (usually >10,000x total coverage) through targeted analysis of a limited pre-selected genomic territory (<500kb). These highly focused cfDNA assay are not well suited for discovery of new resistance mechanisms or the detection of global genetic features such as tumor mutational burden (TMB), which has been shown to be predictive of immunotherapy response [12]. In contrast, broader sequencing assays such as whole exome sequencing (WES, typically >35Mb) are better suited to discovering novel resistance mechanisms [13], quantifying TMB [14], or for characterizing mutational signatures predictive of drug response [15]. However, the sensitivity of plasma WES for detecting individual mutations is generally limited to those mutations that are present at 5% or greater allele frequency given current per megabase sequencing costs. Recent studies have also demonstrated the feasibility of ultra-deep sequencing (>60,000x) across a medium-size panel (~500 genes) to reveal low-allele frequency mutations in plasma [16], or 30X whole genome sequencing of cfDNA to detect minimal residual disease by tumor-guided genotyping [17]. These approaches represent promising noval platforms for discovery research but remain cost-prohibitive for near-term clinical implementation.

Given the tradeoffs inherent in current cfDNA platforms, negative cfDNA results need to be interpreted with caution as the failure to detect a potentially actionable mutation or mutational signature may be due to low tumor fraction in plasma or, in the case of targeted panel sequencing, the presence of driver mutations in genomic loci not covered by the assay design. In this study, we assessed whether cfDNA tumor fraction estimation through low-pass, shallow whole genome sequencing (sWGS) [15, 18, 19], fragment size analysis [20, 21], or both [22] could facilitate the interpretation of negative cfDNA results and guide the choice between broader WES and less comprehensive but more sensitive ultra-deep sequencing assays to screen for clinically relevant mutations or mutational signatures.

## Methods

### Sample collection, consent, and patient characteristics

Patients with metastatic solid tumors treated at a single academic cancer center (Memorial Sloan Kettering Cancer Center, New York, USA) were studied. Patients had one of several tumor types including breast cancer, prostate cancer, urothelial cancer, testicular cancer, melanoma, and non-small cell lung cancer (Additional file 1: Table S1). Patients were consented to an IRB-approved research protocol (NCT01775072) which permits genomic profiling of tumors, cfDNA, and matched normal blood.

### Plasma processing, cfDNA extraction

Whole blood was collected in 10-ml Cell-Free DNA BCT tubes (STRECK, USA) and centrifuged in two steps to separate cell-free supernatant from cells. In step 1, samples were centrifuged at 800g for 10 min (ambient temperature). Plasma supernatant was then separated from red blood cells. In step 2, separated supernatant was further centrifuged in a high-speed micro-centrifuge at 18,000*g* for 10 min (ambient temperature). Cell-free plasma supernatant was then aliquoted and frozen at −80°C until DNA extraction. Extraction of cfDNA was performed using a fully automated QIAGEN platform, QIAsymphony SP, and the QIAsymphony DSP Virus/Pathogen Midi Kit (QIAGEN, Germany). Quality and quantity of cfDNA was evaluated with automated electrophoresis using the Fragment Analyzer with the High Sensitivity genomic DNA Analysis Kit (Advanced Analytical, USA). Plasma samples from 10 healthy donors were subjected to the same extraction and quantification process.

### MSK-IMPACT analysis of tumor and plasma DNA

Two hundred fifty nanograms of DNA extracted from tumor and matched whole blood normal were subjected to targeted sequencing using MSK-IMPACT to a target depth of 644x as previously described [23]. Sequencing libraries were prepared according to the KAPA Hyper protocol (Kapa Biosystems, USA) with the ligation of Illumina sequence adaptors followed by PCR amplification and purification as described [23]. Sample-specific indexes were added to each library. For cf-IMPACT, 5–100 ng of DNA was extracted from plasma or 50 ng DNA from matched white blood cells and then subjected to the same protocol except that an adapter concentration of 4.5 μM was used to increase the reaction efficiency. Pre-capture libraries were quantified with Qubit (Invitrogen, USA). An equal amount of each DNA library (~250 ng per sample) was pooled for hybridization capture using the NimbleGen SeqCap Target Enrichment system (Roche, USA) at 55°C for 16 h, followed by post-capture washes and 16 cycles of PCR amplification. The pooled, purified libraries containing captured DNA fragments were then sequenced using the Illumina HiSeq system to an average of 631x depth. The version of MSK-IMPACT used for cfDNA profiling (cf-IMPACT) was designed to detect known mutations at 1% VAF by genotyping based on prior tumor sequencing results, or *de novo* identification of mutations at 2% (hotspot) and 5% (non-hotspot) allele frequency across 410 genes [23, 24]. When available, matched tumor tissue sequencing results were analyzed to assess the clonality of single nucleotide variants (SNVs) using FACETS (v.0.5.6) [25]: A clonal mutation was defined as a mutation with an estimated cancer cell fraction (CCF) of 75% or higher, and sub-clonal mutations were those with a CCF below 75%. Variant allele frequencies were determined by calculating the ratio of sequencing reads supporting the variant allele versus the total (mutant + wild type) number of reads at a given locus. When multiple mutations were detected, the median variant allele frequencies (mVAF) were calculated. MSI status in tumor and plasma was determined using MSIsensor [26]. Tumor mutational burden (TMB) in plasma samples analyzed by cf-IMPACT was calculated as the number of non-synonymous mutations per megabase (mutation/Mb) based on an panel size of 1,016,478 bp. When comparing the mutation data between tumor and plasma, we focused our analysis on the 410 genes that were covered by both the tumor MSK-IMPACT and plasma cf-IMPACT.

### Shallow whole genome sequencing (sWGS) and whole exome sequencing of plasma cfDNA

sWGS of plasma cfDNA and whole exome sequencing [13] of plasma cfDNA were performed as previously described [27]. Briefly, for sWGS, libraries were sequenced to approximately 10 million reads per sample and analyzed using Plasma-Seq, which generates a genome-wide *z*-score as an estimation of tumor fraction by comparing global copy number alterations in a given plasma sample to a panel of normal healthy donors [18, 27]. Patients were then stratified using the Plasma-Seq algorithm as having high (*z*-score≥5) or low (*z*-score<5) cfDNA tumor fraction [19]. For comparison, the same data were also analyzed using the ichorCNA algorithm to estimate cfDNA tumor fraction [15]. Fragment lengths were collected from the insert size data and short fragments were defined as 0–150 bp and long fragments as 151–500 bp [20]. For whole exome sequencing, sequencing libraries were pooled in appropriate ratios depending on total mass and subjected to exome capture using the NimbleGen SeqCap Exome Target Enrichment system and sequenced to a mean depth of 384X. Somatic alterations and mutational signatures were identified using a custom bioinformatics pipeline (https://github.com/mskcc/mutation-signatures). Clinical actionability of the variants were defined according to the OncoKB Precision Oncology Database [28].

### Ultra-deep sequencing of cfDNA using the MSK-ACCESS assay

Approximately 10 ng of cfDNA or 50 ng of matched buffy coat DNA per sample was used for DNA library construction using the KAPA Hyper DNA library preparation kit (Roche, Switzerland). Custom DNA probes and unique molecular indexes (UMI) (Integrated DNA Technologies, USA) were designed to capture selected exons and introns of 129 genes. Pre-capture libraries were quantified with Qubit (Thermo Fisher Scientific, USA). Equal amounts of each DNA library were then pooled for hybridization capture using a customized capture protocol modified from the NimbleGen SeqCap Target Enrichment system (Roche, Switzerland). The captured DNA libraries were then sequenced on an Illumina HiSeq with paired end reads (2×100 bp). A panel of 10 normal cfDNA samples was analyzed to establish the background error profiles in order to remove sequencing artifacts. Sequencing reads were aligned to the human genome (hg19) after UMI clipping, and reads originating from the same DNA fragments were collapsed into consensus read sequences using a custom collapsing algorithm (https://github.com/mskcc/Marianas). Consensus reads were then aligned back to the human genome followed by variant calling using a custom pipeline involving mutation callers VarDict (V1.5.1) [29] and MuTect (V1) [30]. A summary of the sequencing analysis workflow of the 118 samples in this study is shown in Additional file 2 Fig. S1.

### Statistical analyses

We applied a logistic regression model with 5-fold cross validation to distinguish between high (≥ 10% mVAF) or low (<10% mVAF) tumor fractions in cfDNA, using the following features: (i) copy number profiles (represented by genome-wide z-scores) derived from sWGS and (ii) fragment size profiles from targeted sequencing (cf-IMPACT). We evaluated the performance of predicting tumor fraction using different ranges of fragment size distribution, including 40–140 bp, 163–169 bp, and 210–330 bp extracted from the respective sequencing data, and chose the size range with the best performance to then compare with the copy number-based method. Differences in the fraction of cfDNA in these size regions have been reported to distinguish cancer patients from non-cancer individuals’ cfDNA [20]. The performance of each type of feature was measured by 5-fold cross-validation and then used to plot a receiver operating characteristic (ROC) curve and to calculate the area under the curve (AUC). A Mann-Whitney *U* test was performed to determine the difference in distributions in the genome-wide *z*-scores between different groups. Fisher’s exact test was performed to test for enrichment of agreement between tumor and plasma in different categories. A Pearson’s chi-squared test with Yate’s continuity correction was applied to determine the independence of categorical variables such as the agreement between tumor and plasma and different tumor content. A *p*-value <0.05 was considered statistically significant.

## Results

### Estimation of cfDNA tumor fraction using genome-wide copy number profiles and cfDNA fragment size

**Fig. 1:**
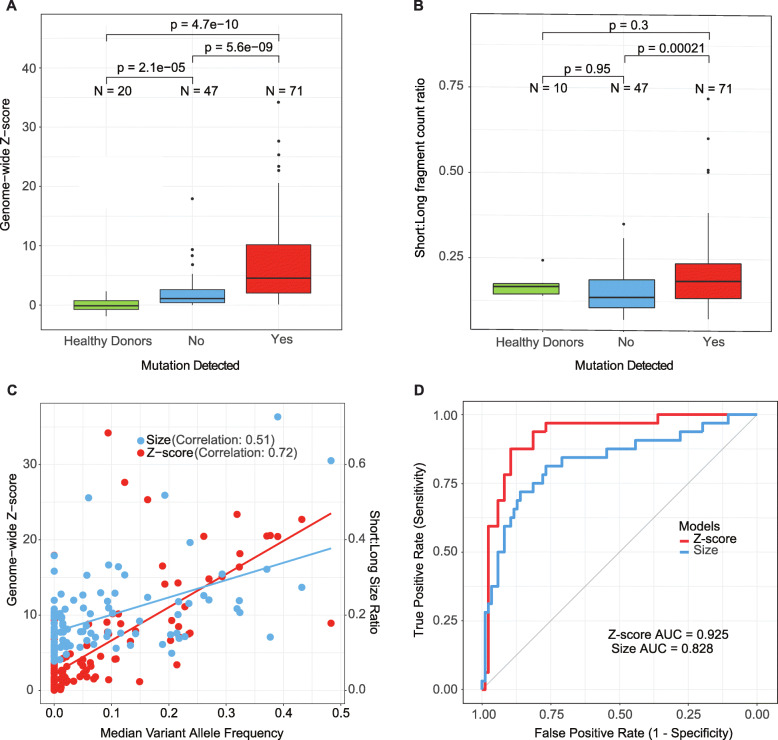
Estimation of cfDNA tumor fraction by genome-wide copy number profiles or fragment size profiles. **a** Comparison of shallow whole genome sequencing (sWGS)-estimated *z*-score distribution between plasma samples from healthy controls and cancer patients with or without mutations detected by cf-IMPACT (cell-free MSK-IMPACT). **b** Comparison of cfDNA fragment size, expressed as the ratio of the counts between short to long fragments (0–150 bp)/(151–500 bp), in plasma samples from healthy controls and cancer patients with or without mutations detected by cf-IMPACT. **c** Correlation between sWGS-estimated *z*-scores and median variant allele fraction (mVAF) as quantitated by cf-IMPACT analysis of plasma cfDNA. Correlation between the ratio of the counts between short to long fragment (0–150 bp)/(151–500 bp) computed from cf-IMPACT data and median variant allele frequency (mVAF) quantitated by cf-IMPACT analysis in cfDNA. **d** Comparison of model performance of global copy number change (*Z*-scores) from sWGS and the short to long fragment size ratio computed from cf-IMPACT data to predict high or low tumor fraction

Plasma was prospectively collected and analyzed from 118 solid tumor patients with progressing metastatic disease. Each plasma sample was analyzed using both cell-free MSK-IMPACT (cf-IMPACT) and shallow whole genome sequencing (sWGS). While cf-IMPACT can detect mutations, copy number alterations, and gene fusions in 410 cancer-associated genes, we focused in this study on the detection of non-synonymous mutations. To establish a rough estimate of tumor fraction in cfDNA, we computed the mean variant allele frequency (mVAF) in each sample based on the mutations detected by cf-IMPACT. In parallel, we used two algorithms, Plasma-Seq and IchorCNA, to generate a tumor fraction estimate by calculating a genome-wide *z*-score based on chromosomal copy number alterations in cfDNA measured by sWGS [15, 27]. We found that the sWGS-based estimated genome-wide *z*-scores were significantly higher in patients with mutations detected by cf-IMPACT (mean 7.91; range 0.106–34.2) compared to those without mutations detected (mean 2.14; range 0.0178–17.9; Mann-Whitney test, *p*=5.6e−09), and healthy blood donors (mean 0.026; range −1.86 to 2.29 Mann-Whitney test, *p*=4.7e−10) (Fig. 1a). These observations held true when tumor fraction was estimated using ichorCNA instead of *z*-score statistics (Additional file 2: Fig. S2A). As the *z*-scores in the healthy donor samples were calculated by comparing the samples to another independent cohort of healthy individuals previously published [27], *z*-scores <0 could be observed due to low-level inter-individual variations. The ichorCNA tumor fraction estimates also strongly correlated with both the mVAF (correlation coefficient 0.84; Additional file 2: Fig. S2B) and the *z*-scores (correlation coefficient = 0.72; Additional file 2: Fig. S2C).

Since the copy number-based approach to estimate cfDNA tumor fraction in plasma is dependent on the presence of tumor-specific copy number alterations, it may underestimate cfDNA tumor fraction in patients with tumors that are copy number neutral. To address this possibility, we evaluated alternative strategies to estimate cfDNA tumor fraction that do not depend on tumor genomic information. Previous studies have shown that the average fragment length of tumor-derived cfDNA is shorter than cfDNA derived from normal white blood cells and that the relative proportions of short and long fragment sizes differs between cancer patients and healthy individuals [20]. We therefore evaluated the performance of different fragment size ranges (see the “Methods” section) to predict whether or not a given cfDNA sample would have a mVAF of 10% or greater, using a logistic regression model with 5-fold cross-validation. We found that the ratio of short to long fragment size (0–150 bp)/(151–500 bp) provided the best performance among the size ranges tested. We then computed the ratio of short to long fragment size (0–150 bp)/(151–500 bp) of the cf-IMPACT data and found that samples with mutations detected by cf-IMPACT had a significantly higher ratio of short to long fragments than samples with no mutations detected (Mann-Whitney test, *p*=0.00021, Fig. 1b). We then plotted the distribution of genome-wide *z*-score and short to long size ratio, respectively, against the mVAF, and found a strong correlation between *z*-score and mVAF (correlation coefficient 0.72) but only a modest correlation between size ratio and mVAF (correlation coefficient 0.51, Fig. 1c). We then compared the performance of cfDNA tumor fraction prediction based on fragment size versus sWGS genome-wide *z*-scores and found that copy number-based *z*-score statistics (AUC=0.925) performed better than size-based estimates (AUC=0.828) (Fig. 1d). Combining the two features (fragment size profiles and *z*-scores) resulted in similar performance (AUC=0.928) to that of sWGS-based *z*-score alone. Therefore, in this study, we decided to use the sWGS-based *z*-score alone to estimate whether a cfDNA sample had low or high tumor fraction.

### cf-IMPACT analysis detected tumor-derived mutations in the majority of plasma samples with high tumor fraction

**Fig. 2:**
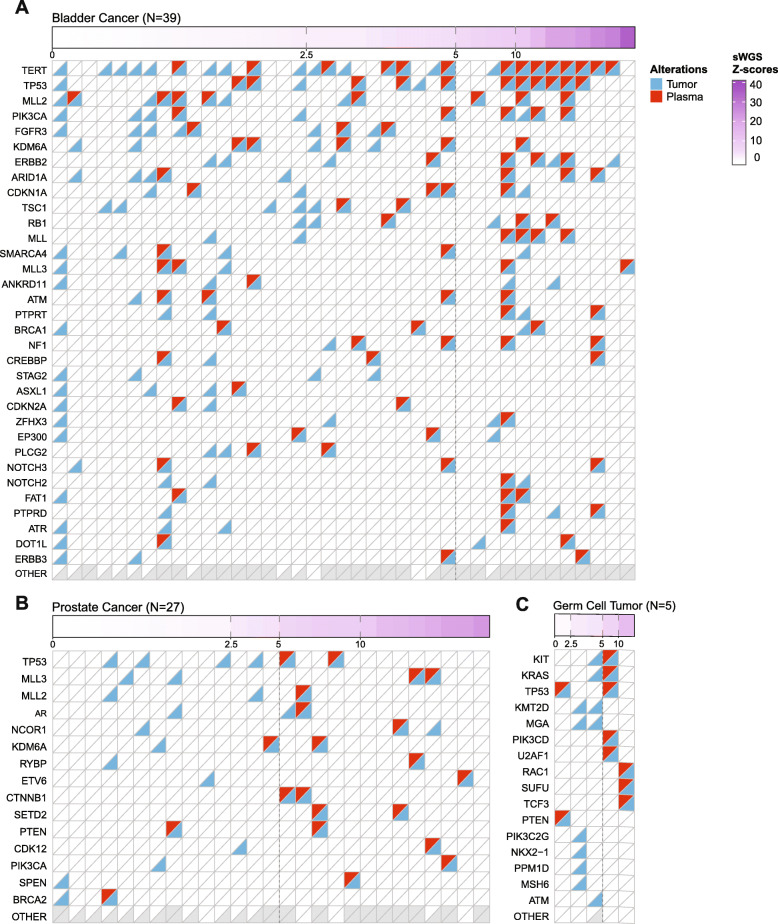
Detection of tumor-derived mutations in plasma by cf-IMPACT as a function of cfDNA tumor fraction. **a**–**c** Concordance of mutations detected by tumor and cf-IMPACT as a function of increasing *z*-scores. Patients with **a** bladder, **b** prostate, and **c** germ cell cancers that had both tumor and plasma mutational data are shown. The top 8% (bladder), 4% (prostate), and 20% (germ cell tumor) most frequently mutated genes are shown. The thresholds of 2.5 and 5 *z*-scores corresponding to 5% and 10% tumor fraction delineates a clear cutoff between a majority of samples with mutations detected in plasma from samples with few or no plasma mutations detected in each of the cancer types

In the 76 patients with available tumor mutation profiling data, we identified somatic mutations in the cfDNA of 72% (55/76) using a combination of de novo mutation identification and genotyping of previously known mutations from the patient-matched tumor MSK-IMPACT results. cfDNA samples with at least one mutation detected had significantly higher genome-wide *z*-scores compared to those patients with no mutations detected in plasma (*p*-value = 1.2e−05) and unrelated healthy blood donors (*p*-value=0.0002) (Additional file 2: Fig. S3A). We next compared the distribution of *z*-scores to the mVAF and found that 22 (88%) of the 25 samples with a mVAF of ≥10% had a *z*-score of ≥5, and 46 (90%) of the 51 samples with mVAF of <10% had *z*-scores of <5 (Additional file 2: Fig. S3B), consistent with published results [8]. We also found that the percentage of all tumor mutations that were detected in plasma was significantly higher in plasma samples with *z*-scores of 5 or higher (Mann-Whitney test, *p*=3.3e−07; Fig. 2, Additional file 2: Fig. S4). Notably, in some patients, cfDNA analysis also revealed sub-clonal mutations that were present in the tumor below the detection threshold of the MSK-IMPACT tumor profiling assay. For example, in a metastatic castration-resistant prostate cancer (mCRPC) patient, cf-IMPACT analysis of plasma revealed an *AR* p.H875Y mutation, a likely acquired resistance mechanism to prior hormonal therapy. A tumor biopsy was then collected 6 days after the cfDNA sample, and MSK-IMPACT analysis confirmed the presence of this *AR* mutation at a VAF of 0.3%, significantly below the threshold for de novo mutation calling using the MSK-IMPACT assay. These data are consistent with prior studies suggesting the potential for cfDNA to detect clinically informative sub-clonal mutations [9, 31].

### High cfDNA tumor fraction was associated with mutational concordance between cfDNA and tumor mutational profiles

**Fig. 3:**
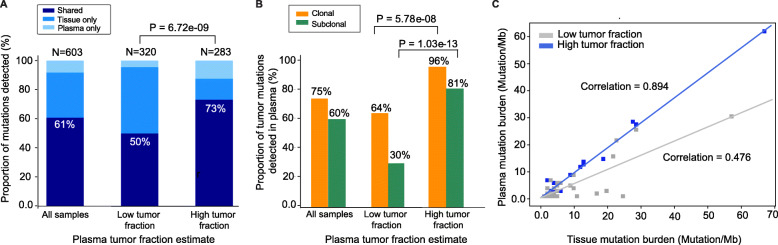
Agreement between plasma and tumor MSK-IMPACT profiles in the context of sWGS-estimated cfDNA tumor fraction. **a** Comparison of the proportion of mutations detected in both plasma and tumor (shared, percentages shown on graph), versus mutations detected in tumor only, or plasma only. Data shown for three categories: all samples, samples with low tumor fraction (*z*-score <5) in plasma, and samples with high tumor fraction (*z*-score ≥5) in plasma. **b** Comparison of the proportion of clonal versus subclonal tumor mutation detected in plasma samples. Data shown for three categories: all samples, samples with low tumor fraction (*z*-score <5) in plasma, and samples with high tumor fraction (*z*-score ≥5) in plasma. Clonality was defined based on tumor cancer cell fractions estimated by FACETS analysis. **c** Comparison of mutation burden as quantitated by MSK-IMPACT analysis of tumor and plasma. Samples are color coded based on *z*-score: ≥5 (blue) versus <5 (gray)

We then investigated the mutational concordance between tumor and plasma mutational profiles in the context of (1) sWGS-based *z*-score (as an estimate of high versus low tumor fraction in cfDNA), (2) clonality of the mutations in the corresponding tumor, (3) the time interval between tumor and plasma collection, and (4) whether the mutation was oncogenic and/or therapeutically actionable. We found that the fraction of shared mutations between tissue and plasma was significantly higher in cfDNA samples with *z*-scores ≥5 (207/283, 73%) relative to those with a *z*-score <5 (160/320, 50%, Fisher’s exact test, *p*-value = 6.72e−09; Fig. 3a, Additional file 3: Table S2). This observation held true for both clonal (70/73, 96% [*z*-score ≥5] versus 97/151, 64% [*z*-score <5], Fisher’s exact test, *p*-value = 5.78e−08) and sub-clonal (99/122, 81% [*z*-score ≥5] versus 25/84, 30% [*z*-score <5], Fisher’s exact test, *p*-value = 1.03e−13) mutations (Fig. 3b). We next evaluated the effect of collection time interval between tumor and plasma. Patients with plasma and tumor samples collected less than 180 days apart generally had a higher, but not statistically significant, median proportion of tumor mutations detected in plasma than those with the two specimens collected 180 days or more apart (Mann-Whitney test, *p*=0.22, Additional file 2: Fig. S5). To account for the effect of *z*-score in this analysis, we confirmed that there was no significant difference in *z*-scores between the two collection time intervals (Mann-Whitney test *p*-value = 0.24). Similarly, mutation type (hotspot, oncogenic, or clinically actionable) was also not associated with the likelihood of detection in plasma (Additional file 2: Fig. S6).

### TMB quantitation and assessment of MSI status by cf-IMPACT

Tumor mutation burden (TMB) and microsatellite instability (MSI) status have been associated with response to immunotherapy [12]. One limitation of current commercial cfDNA assays is that their small genomic footprint (typically <500kb) limits their ability to quantify tumor mutational burden or detect mutational signatures associated with drug response. Among patients with tumor data available, tumor-based MSK-IMPACT analysis identified 15 patients with high TMB (defined as ≥10 mutations/Mb). Of these 15 patients, 11 of them were also found to have ≥10 mutations/Mb in the corresponding cf-IMPACT analysis. The remaining 4 cfDNA samples all had *z*-score <5, suggesting that the lack of TMB concordance between tumor and plasma analysis in these cases was likely due to low levels of tumor-derived DNA in plasma, rather than lesion-to-lesion genomic heterogeneity. The correlation of TMB between matched tumor and plasma was also higher in patients with *z*-scores ≥5 (correlation coefficient between tumor and plasma TMB: 0.894, *p*-value: 1.7e−09, slope = 0.929) versus *z*-scores <5 (correlation coefficient: 0.476, *p*-value: 0.0079, slope = 0.525) (Fig. 3c).

**Fig. 4.**
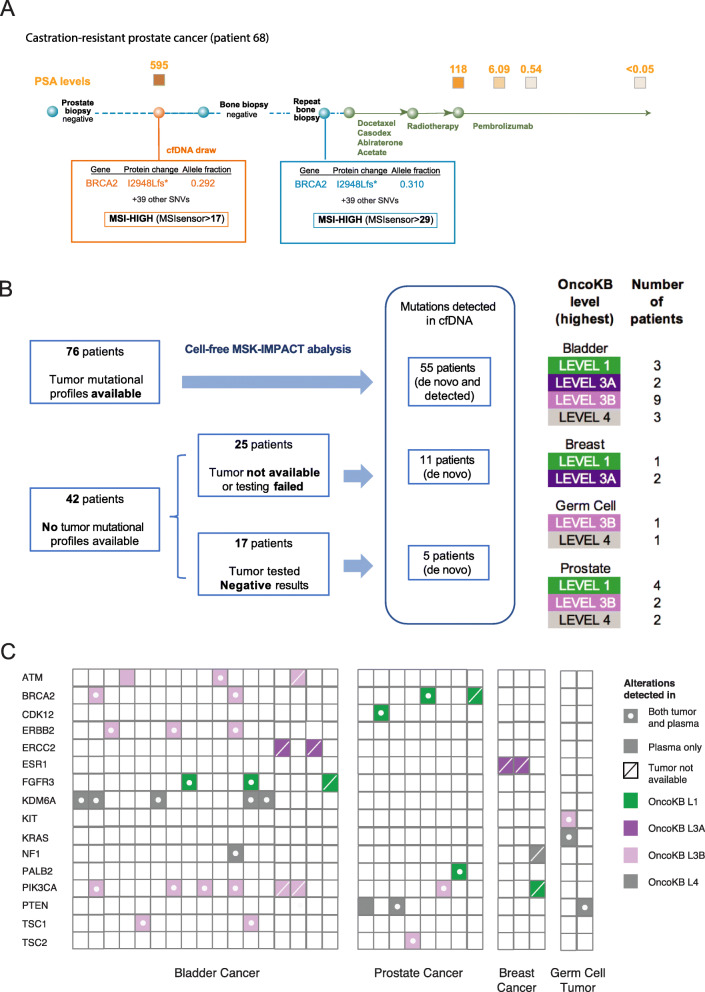
cf-IMPACT revealed actionable alterations in plasma without prior knowledge from tumor. **a** Treatment timeline of a metastatic prostate cancer patient whose initial prostate needle biopsy and bone biopsy showed negative results on tumor MSK-IMPACT testing. cf-IMPACT revealed MSI-High status and a high tumor mutational burden. A later tumor biopsy confirmed these results and the patient was then treated with pembrolizumab resulting in a significant clinical response, as reflected by a sharp drop in serum PSA from 118 to 6 within a month and later to undetectable levels. **b** Summary of the number of patients analyzed by cf-IMPACT and the proportion with somatic variants of potential clinical actionability according to the OncoKB knowledgebase. De novo analysis refers to the identification of mutations without prior knowledge of the tumor mutational profile. Mutations *detected* refers to the genotyping of mutations in cfDNA based on prior knowledge of the matching tumor. **c** Summary of mutations in patients with OncoKB level 1–4 variants (gene name shown) identified in plasma cfDNA. Mutations that were detected in both tumor and plasma are indicated with a dot and a filled square. Mutations detected only in plasma but not in the matched tumor are indicated with a filled square. Mutations detected in plasma in patients for whom tumor analysis was not available are indicated with a filled square with a line. The colors of the boxes represent the corresponding OncoKB annotations (green=level 1, dark purple=level 3A, light purple=level 3B, gray=level 4)

Apart from TMB, we also sought to determine whether MSI status could be accurately determined from cf-IMPACT analysis using MSIsensor [26]. The cohort included two metastatic castration-resistant prostate cancer patients with high MSIsensor scores (>10), both of whom were found to have high TMB based on the plasma cf-IMPACT analysis above. In one patient, at the time of cf-IMPACT analysis, two prior tumor biopsies collected from the prostate and bone had been deemed inadequate for MSK-IMPACT tumor genomic profiling due to insufficient tumor DNA. After detecting the MSI-High status by cf-IMPACT, a subsequent third biopsy (a bone lesion) confirmed the cf-IMPACT result, leading to treatment with the anti-PD-1 antibody pembrolizumab following progression on hormonal therapies [32]. Pembrolizumab treatment resulted in a dramatic and durable response with a decline in serum PSA from 118 to <10, which has been durable for over a year (Fig. 4a).

### Analysis of plasma DNA revealed clinically actionable mutations without prior knowledge from the tumor

A common challenge with tumor-based molecular profiling is the lack of adequate tumor tissue for NGS-based genomic profiling. We therefore sought to determine whether cf-IMPACT could identify targetable genomic alterations in the 42 patients for whom adequate tumor tissue was unavailable for tumor profiling (*N*=25) or in whom prior tumor testing had failed to identify any known oncogenic mutations (*N*=17). In total, cf-IMPACT identified somatic mutations in 11 of 25 (44%) patients who had no tumor available or in whom the test had failed due to poor sample/DNA quality, and 5 of 17 (29%) patients whose tumors had previously be analyzed by MSK-IMPACT but no somatic alterations were identified (Fig. 4b). Mutations detected included OncoKB Level 1 alterations (defined as predictive biomarkers of response to an FDA-approved drug) such as *BRCA2* mutations, which are predictive of response olaparib (a poly(adenosine diphosphate-ribose) polymerase (PARP) inhibitor) in prostate cancer [33], and *PIK3CA* mutations, which are predictive of response to alpelisib (a selective PI3 kinase inhibitor) in breast cancer [34] (Fig. 4c). Consistent with the results in the 76 patients with matched tumor tissue, sWGS-based *z*-scores were significantly higher in the samples with mutations detected as compared to those without mutations detected (Additional file 2: Fig. S3).

Across the entire cohort, cf-IMPACT identified somatic mutations in cfDNA in 71/118 (60%) patients, including variants associated with potential clinical actionability (OncoKB levels 1-4) [28] in 30/118 (25%) patients (Fig. 4c). In the 47 patients in which cf-IMPACT did not detect any somatic variants, 42/47 had *z*-score <5 (Additional file 2: Fig. S1).

### Ultra-sensitive targeted sequencing can identify clinically relevant alterations in plasma samples with low tumor fractions

To explore the biologic basis for the failure to detect any somatic mutations in the plasma samples of the 42 patients with no mutations detected by cf-IMPACT and sWGS-estimated *z*-scores <5, we utilized an ultra-deep sequencing assay (MSK-ACCESS) that could detect mutations at a VAF as low as 0.1%. We hypothesized that a subset of these patients had actionable tumor-derived somatic mutations in plasma at allele frequencies below the limit of detection of cf-IMPACT. To achieve higher sensitivity, we employed error correction using unique molecular indices. As this approach requires significantly greater sequencing depth (target depth of coverage of >12,000x), the breadth of this assay was limited to selected exonic and intronic regions of only 129 cancer associated genes (around 13% of the genomic territory covered by cf-IMPACT).

**Fig. 5:**
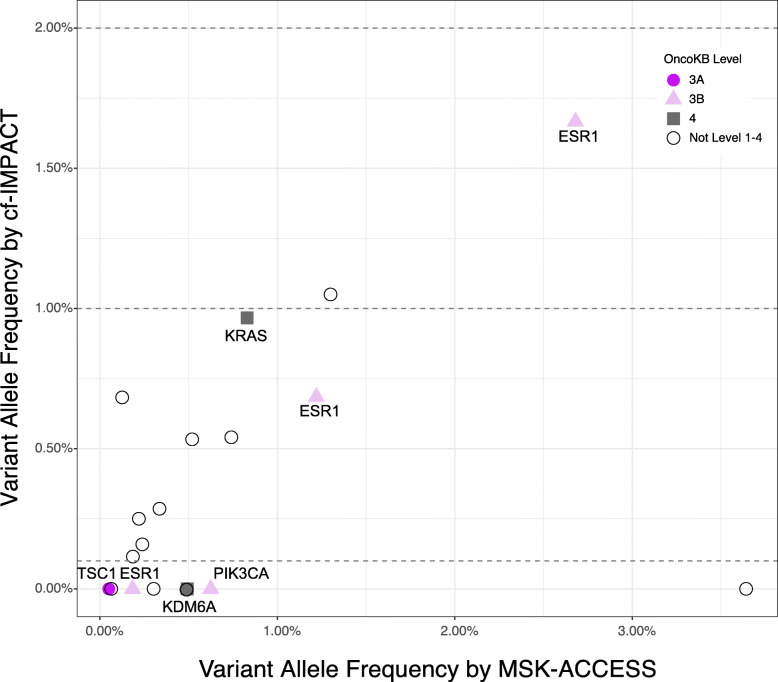
cfDNA tumor fraction guides the optimal selection of profiling assays. **a** MSK-ACCESS analysis of cfDNA samples with sWGS-estimated *z*-score <5 and no mutations detected by cf-IMPACT identified mutations at allele fractions below the detection limit of cf-IMPACT. Mutations with potential clinical relevance that were not detected by cf-IMPACT but were identified by MSK-ACCESS are highlighted. Retrospective manual curation of cf-IMPACT data guided by MSK-ACCESS results revealed evidence of a subset of mutations below the detection limit of cf-IMPACT. The dotted lines indicate the two different detection limits of cf-IMPACT: 1% for genotyping of mutation known from tumor profling and 2% for de novo calling of hotspot mutations. The colors of the shapes represent the corresponding OncoKB annotations (dark purple=level 3A, light purple=level 3B, gray=level 4, open = variants not listed on levels 1–4)

Of the 42 patients with no mutations detected by cf-IMPACT and *z*-scores <5, 29 had sufficient leftover plasma derived DNA for analysis by MSK-ACCESS. Within this subset, MSK-ACCESS identified 19 high-confidence somatic mutations in 14 (48%) patients. These mutations had a median VAF of 0.49% (range 0.05–3.64%), and 7 (34%) were clinically actionable based on the OncoKB knowledgebase [28] (Fig. 5, Additional file 4: Table S3). A notable example was a heavily pre-treated metastatic breast cancer patient in which neither tumor (MSK-IMPACT) nor cf-IMPACT detected any somatic mutations. Ultra-deep sequencing of cfDNA using the MSK-ACCESS assay, identified an *ESR1* p.E380Q mutation, an alteration previously associated with resistance to hormonal therapy [35], at a variant allele frequency of 1.7%. Notably, evidence of this mutation was present in the cf-IMPACT data below the detection threshold of that assay, illustrating that more sensitive profiling methods could identify alterations of potential clinical relevance in samples with low tumor fraction.

### Whole exome sequencing of plasma samples with high cfDNA tumor fraction to identify tumor-derived mutational signatures and oncogenic alterations

Five of the 47 patients with no mutations detected by cf-IMPACT had sWGS-estimated *z*-scores ≥5. We hypothesized that these samples harbored oncogenic mutations in genes not covered by the MSK-IMPACT panel. Indeed, WES of cfDNA (cf-WES) identified somatic mutations in all 5 samples (average 212, range 169–268 mutations) with an average mVAF of 11% (range 8.1–13.4%). Ninety-nine percent of mutations identified by cf-WES were in genomic regions not covered by MSK-IMPACT, with 13.1% of the mutations present in genes reported to be mutated in the TCGA analyses of the respective cancer types. We were able to obtain sufficient tumor material to perform WES on the tumor specimens from 3 of the 5 patients who underwent cf-WES and observed that the predominant mutational signatures found in tumor were also detectable in plasma. cf-WES also revealed likely oncogenic alterations not covered by the cf-IMPACT assay design including a likely oncogenic frameshift deletion in the tumor suppressor *IRF1* [36] in a prostate cancer patient, and in a urothelial cancer patient a likely oncogenic frameshift deletion in *EP400*, which encodes a component of the NuA4 histone acetyltransferase complex that positively regulates transcription [37]. These results confirm that cfDNA tumor fraction estimates based on sWGS-based *z*-scores can identify patients who could benefit from a more comprehensive plasma-based profiling approaches.

Taken together, tumor fraction-guided ultra-deep or whole-exome sequencing identified oncogenic or likely oncogenic mutations in 19/34 (43%) samples with negative results by cf-IMPACT. Overall, using the three complementary plasma profiling approaches, we identified mutations in 90/118 (76%) patients in the entire cohort.

## Discussion

Tumor molecular profiling is increasingly used to guide treatment selection in patients with advanced solid tumors. Oncologists now need to rapidly screen for an increasing number of disease-specific or tumor agnostic biomarkers of drug response, and a lack of adequate tumor tissue for comprehensive tumor profiling can delay the administration of the most appropriate systemic therapies. Patients with metastatic cancers that are difficult to biopsy, such as bone only metastatic prostate and breast cancers, are at particular risk of never receiving the most effective targeted therapies or potentially curative immunotherapies [38].

The observation that tumor-derived DNA is present in the plasma of patients with cancer has made possible the non-invasive detection of actionable somatic mutations as a guide to treatment selection [39]. While whole exome and genome-scale sequencing of cfDNA is feasible in cancer patients [13, 17], the low fraction of cfDNA derived from tumor and the high cost of sequencing limit the clinical feasibility of such approaches. Conversely, more sensitive but more focused cfDNA platforms can only detect those clinically actionable mutations covered by the assay design. Small gene panels are also not well suited for the characterization of global genomic features such as mutational signatures or tumor mutational burden, which was recently recognized by the FDA as a tumor agnostic biomarker of immune checkpoint inhibitor response. In the clinical setting, parallel or sequential testing of cfDNA using complementary assays could provide additional clinically relevant information. For example, in cancer types such as ovarian and prostate cancer where targetable hotspot mutations are less common, a broader profiling approach can reveal patterns of structural somatic alteration that are predictive of response to systemic therapies, such as PARP inhibitors or immune checkpoint blockade [40].

In this study, we assessed whether cfDNA tumor fraction estimates could serve as a guide to the interpretation of plasma cfDNA results, especially negative results, and inform clinical decision making. A commonly used method to estimate tumor fraction in plasma DNA is the median variant allele frequencies (mVAF) of multiple mutations determined by sequencing analysis. However, the observed allele frequency of a given mutation can be affected by multiple factors such as copy number changes at the respective genomic loci, loss of heterogeneity or the overall ploidy of the tumor. More importantly, the calculation of mVAF depends heavily on the number of mutations identified by the assay, which is governed by the assay design and size of the panel. A mutation-agnostic approach to quantifying cfDNA tumor fraction could potentially overcome these limitations. In this study, we compared two methods for estimation of cfDNA tumor fraction in a given plasma sample: analysis of genome-wide copy number profiles derived from shallow whole genome sequencing (sWGS), and cfDNA fragment size profiles extracted from targeted sequencing data. These two approaches proved to be complementary: genome-wide copy number estimates were more predictive but not always informative in tumors with few copy number alterations, which accounted for up to 30–40% of the tumor samples in this cohort and in the reported literature [24, 41]. In contrast, fragment size profiles of plasma DNA can be calculated independent of the genomic features of the underlying tumor.

As expected, the overall concordance between tumor and plasma genomic profiles (mutations and TMB estimates) proved to be higher in plasma samples with high cfDNA tumor fraction suggesting that an estimate of cfDNA tumor fraction could help clinicians interpret the robustness of clinical cfDNA profiling results. Of particular note, cfDNA tumor fraction could be used to inform the interpretation of “negative” cfDNA-based genomic profiling results. In cases where no mutations were identified in plasma, cfDNA tumor fraction could help distinguish between samples in which low shedding of DNA from tumor led to a false-negative result from samples in which oncogenic drivers were not detected as they were not covered by the targeted assay design. Furthermore, we were able to use the cfDNA tumor fraction estimates to guide the choice of the most suitable subsequent genomic profiling assay for a given sample: whole exome sequencing to identify mutations not included within the targeted panel or global genomic features for samples with a high cfDNA tumor fraction, or a more focused but more sensitive assay capable of detecting clinically actionable mutations present at low VAF in cfDNA samples with low tumor fraction. We believe that this strategy will be of widespread interest as cfDNA profiling will likely become the initial tumor sequencing assay for many patients with lung and several other cancer types given its relatively shorter turnaround time and the need to rapidly identify actionable drug targets prior to therapy initiation.

The current study had several limitations: Plasma and tumor samples were collected retrospectively, and there was variability in the clinical status of the patients, the time interval between tumor and plasma collections, and the treatment modalities received. Future disease specific analyses may also find that the predictive value of cfDNA tumor fraction estimates based on sWGS or fragment size analysis vary as a function of tumor type. Prospective disease-specific studies incorporating estimation of cfDNA tumor fraction at various stages of disease progression will therefore be needed to evaluate the utility of this approach for disease monitoring and to guide additional diagnostic testing, in particular in patients with no tumor tissue available and negative cfDNA results from targeted panels.

## Conclusions

The results of this study suggest that estimation of cfDNA tumor fraction can facilitate the interpretation of cfDNA results and help guide the selection of the most appropriate alternative assays in patients with negative results. In a prospective setting, this approach could be used to triage samples for cfDNA profiling assays that provide the most appropriate genomic breadth and depth based on the estimated tumor fraction of an individual blood sample.

## Supplementary Information

**Additional file 1: Table S1**. Summary of the tumor types, input cfDNA for cf-IMPACT, and the time between collection of matched tumor and plasma of the 118 patients.

**Additional file 2: Fig. S1**. Summary of the cell-free DNA (cfDNA) profiling assays and number of samples involved in the study. **Fig. S2**. Evaluation of the tumor fraction estimate determined by ichorCNA in cfDNA of healthy controls and cancer patients. **Fig. S3**. Distribution of genome-wide z-scores in patients with and without mutations detected by cfMSK-IPMACT. **Fig. S4**. Comparison of proportion of tumor mutations detected in plasma with z-scores ≥5 and <5. **Fig. S5**. Comparison of proportion of tumor mutations detected in plasma with tumor and plasma collected at different intervals. **Fig. S6**. Proportion of different categories of tumor mutations detected in the corresponding plasma sample.

**Additional file 3: Table S2**. Summary of the mutations found in the tumor and plasma of the 118 patients.

**Additional file 4: Table S3**. Summary of the mutations identified in cfDNA with z-scores <5 using MSK-ACCESS.

## Data Availability

The whole genome sequencing and exome sequencing data from the current study have been deposited in the NCBI’s database of Genotypes and Phenotypes (dbGaP), accession number: phs002290.v1.p1 [42], https://www.ncbi.nlm.nih.gov/projects/gap/cgi-bin/study.cgi?study_id=phs002290.v1.p1. Additional de-identified processed data are available at MSKCC cBioPortal [43]: https://cbioportal.org/study/summary?id=mixed_cfdna_msk_2020

## References

[1] Merker JD, Oxnard GR, Compton C, Diehn M, Hurley P, Lazar AJ, Lindeman N, Lockwood CM, Rai AJ, Schilsky RL, Tsimberidou AM, Vasalos P, Billman BL, Oliver TK, Bruinooge SS, Hayes DF, Turner NC (2018). Circulating tumor DNA analysis in patients with cancer: American Society of Clinical Oncology and College of American Pathologists Joint Review. J Clin Oncol..

[2] Siravegna G, Marsoni S, Siena S, Bardelli A (2017). Integrating liquid biopsies into the management of cancer. Nat Rev Clin Oncol.

[3] Phallen J, Leal A, Woodward BD, Forde PM, Naidoo J, Marrone KA, et al. Early noninvasive detection of response to targeted therapy in non-small cell lung cancer. Cancer Res.2019;79(6):1204–13. 10.1158/0008-5472.CAN-18-1082. 10.1158/0008-5472.CAN-18-1082 PMC6481620 30573519

[4] Chaudhuri AA, Chabon JJ, Lovejoy AF, Newman AM, Stehr H, Azad TD, Khodadoust MS, Esfahani MS, Liu CL, Zhou L, Scherer F, Kurtz DM, Say C, Carter JN, Merriott DJ, Dudley JC, Binkley MS, Modlin L, Padda SK, Gensheimer MF, West RB, Shrager JB, Neal JW, Wakelee HA, Loo BW, Alizadeh AA, Diehn M (2017). Early detection of molecular residual disease in localized lung cancer by circulating tumor DNA profiling. Cancer Discov..

[5] Cohen JD, Li L, Wang Y, Thoburn C, Afsari B, Danilova L, Douville C, Javed AA, Wong F, Mattox A, Hruban RH, Wolfgang CL, Goggins MG, Dal Molin M, Wang TL, Roden R, Klein AP, Ptak J, Dobbyn L, Schaefer J, Silliman N, Popoli M, Vogelstein JT, Browne JD, Schoen RE, Brand RE, Tie J, Gibbs P, Wong HL, Mansfield AS, Jen J, Hanash SM, Falconi M, Allen PJ, Zhou S, Bettegowda C, Diaz LA, Tomasetti C, Kinzler KW, Vogelstein B, Lennon AM, Papadopoulos N (2018). Detection and localization of surgically resectable cancers with a multi-analyte blood test. Science..

[6] Chan KCA, Woo JKS, King A, Zee BCY, Lam WKJ, Chan SL, Chu SWI, Mak C, Tse IOL, Leung SYM, Chan G, Hui EP, Ma BBY, Chiu RWK, Leung SF, van Hasselt AC, Chan ATC, Lo YMD (2017). Analysis of plasma Epstein-Barr virus DNA to screen for nasopharyngeal cancer. N Engl J Med..

[7] Lui YY, Chik KW, Chiu RWK, Ho CY, Lam CW, Lo YMD (2002). Predominant hematopoietic origin of cell-free DNA in plasma and serum after sex-mismatched bone marrow transplantation. Clin Chem..

[8] Bettegowda C, Sausen M, Leary RJ, Kinde I, Wang Y, Agrawal N (2014). Detection of circulating tumor DNA in early- and late-stage human malignancies. Sci Transl Med.

[9] Abbosh C, Birkbak NJ, Wilson GA, Jamal-Hanjani M, Constantin T, Salari R (2017). Phylogenetic ctDNA analysis depicts early-stage lung cancer evolution. Nature..

[10] Tug S, Helmig S, Deichmann ER, Schmeier-Jurchott A, Wagner E, Zimmermann T (2015). Exercise-induced increases in cell free DNA in human plasma originate predominantly from cells of the haematopoietic lineage. Exerc Immunol Rev..

[11] Risberg B, Tsui DWY, Biggs H, Ruiz-Valdepenas Martin de Almagro A, Dawson SJ, Hodgkin C (2018). Effects of collection and processing procedures on plasma circulating cell-free DNA from cancer patients. J Mol Diagn..

[12] Samstein RM, Lee CH, Shoushtari AN, Hellmann MD, Shen R, Janjigian YY, Barron DA, Zehir A, Jordan EJ, Omuro A, Kaley TJ, Kendall SM, Motzer RJ, Hakimi AA, Voss MH, Russo P, Rosenberg J, Iyer G, Bochner BH, Bajorin DF, al-Ahmadie HA, Chaft JE, Rudin CM, Riely GJ, Baxi S, Ho AL, Wong RJ, Pfister DG, Wolchok JD, Barker CA, Gutin PH, Brennan CW, Tabar V, Mellinghoff IK, DeAngelis LM, Ariyan CE, Lee N, Tap WD, Gounder MM, D’Angelo SP, Saltz L, Stadler ZK, Scher HI, Baselga J, Razavi P, Klebanoff CA, Yaeger R, Segal NH, Ku GY, DeMatteo RP, Ladanyi M, Rizvi NA, Berger MF, Riaz N, Solit DB, Chan TA, Morris LGT (2019). Tumor mutational load predicts survival after immunotherapy across multiple cancer types. Nat Genet..

[13] Murtaza M, Dawson S-J, Tsui DWY, Gale D, Forshew T, Piskorz AM, Parkinson C, Chin SF, Kingsbury Z, Wong ASC, Marass F, Humphray S, Hadfield J, Bentley D, Chin TM, Brenton JD, Caldas C, Rosenfeld N (2013). Non-invasive analysis of acquired resistance to cancer therapy by sequencing of plasma DNA. Nature..

[14] Koeppel F, Blanchard S, Jovelet C, Genin B, Marcaillou C, Martin E, Rouleau E, Solary E, Soria JC, André F, Lacroix L (2017). Whole exome sequencing for determination of tumor mutation load in liquid biopsy from advanced cancer patients. Plos One..

[15] Adalsteinsson VA, Ha G, Freeman SS, Choudhury AD, Stover DG, Parsons HA, Gydush G, Reed SC, Rotem D, Rhoades J, Loginov D, Livitz D, Rosebrock D, Leshchiner I, Kim J, Stewart C, Rosenberg M, Francis JM, Zhang CZ, Cohen O, Oh C, Ding H, Polak P, Lloyd M, Mahmud S, Helvie K, Merrill MS, Santiago RA, O’Connor EP, Jeong SH, Leeson R, Barry RM, Kramkowski JF, Zhang Z, Polacek L, Lohr JG, Schleicher M, Lipscomb E, Saltzman A, Oliver NM, Marini L, Waks AG, Harshman LC, Tolaney SM, van Allen EM, Winer EP, Lin NU, Nakabayashi M, Taplin ME, Johannessen CM, Garraway LA, Golub TR, Boehm JS, Wagle N, Getz G, Love JC, Meyerson M (2017). Scalable whole-exome sequencing of cell-free DNA reveals high concordance with metastatic tumors. Nat Commun..

[16] Razavi P, Li BT, Brown DN, Jung B, Hubbell E, Shen R, Abida W, Juluru K, de Bruijn I, Hou C, Venn O, Lim R, Anand A, Maddala T, Gnerre S, Vijaya Satya R, Liu Q, Shen L, Eattock N, Yue J, Blocker AW, Lee M, Sehnert A, Xu H, Hall MP, Santiago-Zayas A, Novotny WF, Isbell JM, Rusch VW, Plitas G, Heerdt AS, Ladanyi M, Hyman DM, Jones DR, Morrow M, Riely GJ, Scher HI, Rudin CM, Robson ME, Diaz LA, Solit DB, Aravanis AM, Reis-Filho JS (2019). High-intensity sequencing reveals the sources of plasma circulating cell-free DNA variants. Nat Med..

[17] Zviran A, Schulman RC, Shah M, Hill STK, Deochand S, Khamnei CC, et al. Genome-wide cell-free DNA mutational integration enables ultra-sensitive cancer monitoring. Nat Med. 2020;26(7):1114–24. 10.1038/s41591-020-0915-3. Epub 2020 Jun 1. 10.1038/s41591-020-0915-3 PMC8108131 32483360

[18] Heitzer E, Auer M, Hoffmann EM, Pichler M, Gasch C, Ulz P, Lax S, Waldispuehl-Geigl J, Mauermann O, Mohan S, Pristauz G, Lackner C, Höfler G, Eisner F, Petru E, Sill H, Samonigg H, Pantel K, Riethdorf S, Bauernhofer T, Geigl JB, Speicher MR (2013). Establishment of tumor-specific copy number alterations from plasma DNA of patients with cancer. Int J Cancer..

[19] Belic J, Koch M, Ulz P, Auer M, Gerhalter T, Mohan S, Fischereder K, Petru E, Bauernhofer T, Geigl JB, Speicher MR, Heitzer E (2015). Rapid identification of plasma DNA samples with increased ctDNA levels by a modified FAST-SeqS approach. Clin Chem..

[20] Mouliere F, Chandrananda D, Piskorz AM, Moore EK, Morris J, Ahlborn LB, et al. Enhanced detection of circulating tumor DNA by fragment size analysis. Sci Transl Med. 2018;10(466):eaat4921. 10.1126/scitranslmed.aat4921. 10.1126/scitranslmed.aat4921 PMC6483061 30404863

[21] Yu SC, Chan KC, Zheng YW, Jiang P, Liao GJ, Sun H (2014). Size-based molecular diagnostics using plasma DNA for noninvasive prenatal testing. Proc Natl Acad Sci USA..

[22] Cristiano S, Leal A, Phallen J, Fiksel J, Adleff V, Bruhm DC, Jensen SØ, Medina JE, Hruban C, White JR, Palsgrove DN, Niknafs N, Anagnostou V, Forde P, Naidoo J, Marrone K, Brahmer J, Woodward BD, Husain H, van Rooijen KL, Ørntoft MBW, Madsen AH, van de Velde CJH, Verheij M, Cats A, Punt CJA, Vink GR, van Grieken NCT, Koopman M, Fijneman RJA, Johansen JS, Nielsen HJ, Meijer GA, Andersen CL, Scharpf RB, Velculescu VE (2019). Genome-wide cell-free DNA fragmentation in patients with cancer. Nature..

[23] Cheng DT, Mitchell TN, Zehir A, Shah RH, Benayed R, Syed A, Chandramohan R, Liu ZY, Won HH, Scott SN, Brannon AR, O'Reilly C, Sadowska J, Casanova J, Yannes A, Hechtman JF, Yao J, Song W, Ross DS, Oultache A, Dogan S, Borsu L, Hameed M, Nafa K, Arcila ME, Ladanyi M, Berger MF (2015). Memorial Sloan Kettering-Integrated Mutation Profiling of Actionable Cancer Targets (MSK-IMPACT): a hybridization capture-based next-generation sequencing clinical assay for solid tumor molecular oncology. J Mol Diagn..

[24] Zehir A, Benayed R, Shah RH, Syed A, Middha S, Kim HR, Srinivasan P, Gao J, Chakravarty D, Devlin SM, Hellmann MD, Barron DA, Schram AM, Hameed M, Dogan S, Ross DS, Hechtman JF, DeLair DF, Yao JJ, Mandelker DL, Cheng DT, Chandramohan R, Mohanty AS, Ptashkin RN, Jayakumaran G, Prasad M, Syed MH, Rema AB, Liu ZY, Nafa K, Borsu L, Sadowska J, Casanova J, Bacares R, Kiecka IJ, Razumova A, Son JB, Stewart L, Baldi T, Mullaney KA, al-Ahmadie H, Vakiani E, Abeshouse AA, Penson AV, Jonsson P, Camacho N, Chang MT, Won HH, Gross BE, Kundra R, Heins ZJ, Chen HW, Phillips S, Zhang H, Wang J, Ochoa A, Wills J, Eubank M, Thomas SB, Gardos SM, Reales DN, Galle J, Durany R, Cambria R, Abida W, Cercek A, Feldman DR, Gounder MM, Hakimi AA, Harding JJ, Iyer G, Janjigian YY, Jordan EJ, Kelly CM, Lowery MA, Morris LGT, Omuro AM, Raj N, Razavi P, Shoushtari AN, Shukla N, Soumerai TE, Varghese AM, Yaeger R, Coleman J, Bochner B, Riely GJ, Saltz LB, Scher HI, Sabbatini PJ, Robson ME, Klimstra DS, Taylor BS, Baselga J, Schultz N, Hyman DM, Arcila ME, Solit DB, Ladanyi M, Berger MF (2017). Mutational landscape of metastatic cancer revealed from prospective clinical sequencing of 10,000 patients. Nat Med..

[25] Shen R, Seshan VE (2016). FACETS: allele-specific copy number and clonal heterogeneity analysis tool for high-throughput DNA sequencing. Nucleic Acids Res..

[26] Niu B, Ye K, Zhang Q, Lu C, Xie M, McLellan MD (2014). MSIsensor: microsatellite instability detection using paired tumor-normal sequence data. Bioinformatics..

[27] Heitzer E, Ulz P, Belic J, Gutschi S, Quehenberger F, Fischereder K, Benezeder T, Auer M, Pischler C, Mannweiler S, Pichler M, Eisner F, Haeusler M, Riethdorf S, Pantel K, Samonigg H, Hoefler G, Augustin H, Geigl JB, Speicher MR (2013). Tumor-associated copy number changes in the circulation of patients with prostate cancer identified through whole-genome sequencing. Genome Med..

[28] Chakravarty D, Gao J, Phillips S, Kundra R, Zhang H, Wang J (2017). OncoKB: a precision oncology knowledge base. JCO Precision Oncol..

[29] Markovets A, Dougherty B, Barrett JC, Dry JR, Johnson J, Lai Z (2016). VarDict: a novel and versatile variant caller for next-generation sequencing in cancer research. Nucleic Acids Res.

[30] Cibulskis K, Lawrence MS, Carter SL, Sivachenko A, Jaffe D, Sougnez C, Gabriel S, Meyerson M, Lander ES, Getz G (2013). Sensitive detection of somatic point mutations in impure and heterogeneous cancer samples. Nat Biotechnol..

[31] Romanel A, Gasi Tandefelt D, Conteduca V, Jayaram A, Casiraghi N, Wetterskog D (2015). Plasma AR and abiraterone-resistant prostate cancer. Sci Transl Med.

[32] Abida W, Cheng ML, Armenia J, Middha S, Autio KA, Rathkopf DE (2018). Microsatellite instability in prostate cancer and response to immune checkpoint blockade. J Clin Oncol.

[33] de Bono J, Mateo J, Fizazi K, Saad F, Shore N, Sandhu S, Chi KN, Sartor O, Agarwal N, Olmos D, Thiery-Vuillemin A, Twardowski P, Mehra N, Goessl C, Kang J, Burgents J, Wu W, Kohlmann A, Adelman CA, Hussain M (2020). Olaparib for metastatic castration-resistant prostate cancer. N Engl J Med..

[34] Andre F, Ciruelos E, Rubovszky G, Campone M, Loibl S, Rugo HS (2019). Alpelisib for PIK3CA-mutated, hormone receptor-positive advanced breast cancer. N Engl J Med..

[35] Chandarlapaty S, Chen D, He W, Sung P, Samoila A, You D, Bhatt T, Patel P, Voi M, Gnant M, Hortobagyi G, Baselga J, Moynahan ME (2016). Prevalence of ESR1 mutations in cell-free DNA and outcomes in metastatic breast cancer: a secondary analysis of the BOLERO-2 clinical trial. JAMA Oncol..

[36] Bachmann SB, Frommel SC, Camicia R, Winkler HC, Santoro R, Hassa PO (2014). DTX3L and ARTD9 inhibit IRF1 expression and mediate in cooperation with ARTD8 survival and proliferation of metastatic prostate cancer cells. Mol Cancer.

[37] Wu S, Yang Z, Ye R, An D, Li C, Wang Y (2015). Novel variants in MLL confer to bladder cancer recurrence identified by whole-exome sequencing. Oncotarget..

[38] Abida W, Cheng ML, Armenia J, Middha S, Autio KA, Vargas HA, Rathkopf D, Morris MJ, Danila DC, Slovin SF, Carbone E, Barnett ES, Hullings M, Hechtman JF, Zehir A, Shia J, Jonsson P, Stadler ZK, Srinivasan P, Laudone VP, Reuter V, Wolchok JD, Socci ND, Taylor BS, Berger MF, Kantoff PW, Sawyers CL, Schultz N, Solit DB, Gopalan A, Scher HI (2019). Analysis of the prevalence of microsatellite instability in prostate cancer and response to immune checkpoint blockade. JAMA Oncol..

[39] Wan JC, Massie C, Garcia-Corbacho J, Mouliere F, Brenton JD, Caldas C (2017). Liquid biopsies come of age: towards implementation of circulating tumour DNA. Nat Rev Cancer..

[40] Gonzalez-Martin A, Monk BJ (2020). PARP inhibitors in ovarian cancer. Reply. N Engl J Med..

[41] Ciriello G, Miller ML, Aksoy BA, Senbabaoglu Y, Schultz N, Sander C (2013). Emerging landscape of oncogenic signatures across human cancers. Nat Genet..

[42] Tsui DWY, Cheng ML, Shady M, Yang JL, Stephens D, Won H, et al. Tumor fraction guided cell-free DNA profiling in metastatic cancer patients. dbGAP. 5 Feb 2020; https://www.ncbi.nlm.nih.gov/projects/gap/cgi-bin/study.cgi?study_id=phs002290.v1.p1 10.1186/s13073-021-00898-8 PMC8165771 34059130

[43] Tsui DWY, Cheng ML, Shady M, Yang JL, Stephens D, Won H, et al. Mixed cfDNA (MSKCC, 2020). cBioPortal. 5 Feb 2020; https://www.cbioportal.org/study/summary?id=mixed_cfdna_msk_2020

