# Supplementary material for: Tumor fraction-guided cell-free DNA profiling in metastatic solid tumor patients
Source: Genome Med. 2021 May 31;13:96. doi: 10.1186/s13073-021-00898-8 (PMC8165771; doi:10.1186/s13073-021-00898-8)
Supplement: Supplementary file 2 — Additional file 2: Fig. S1. Summary of the cell-free DNA (cfDNA) profiling assays and number of samples involved in the study. Fig. S2. Evaluation of the tumor fraction estimate determined by ichorCNA in cfDNA of healthy controls and cancer patients. Fig. S3. Distribution of genome-wide z-scores in patients with and without mutations detected by cfMSK-IPMACT. Fig. S4. Comparison of proportion of tumor mutations detected in plasma with z-scores ≥5 and <5. Fig. S5. Comparison of proportion of tumor mutations detected in plasma with tumor and plasma collected at different intervals. Fig. S6. Proportion of different categories of tumor mutations detected in the corresponding plasma sample. [file 13073_2021_898_MOESM2_ESM.docx]

**Additional file 2**

**Additional file 2: Fig. S1**

Summary of the cell-free DNA (cfDNA) profiling assays and number of samples involved in the study.

118 cfDNA samples were analyzed by cf-IMPACT and sWGS (shallow whole genome sequencing). cfDNA with no mutations detected where then further analyzed by either MSK-ACCESS (whenever samples available) or whole exome sequencing based on tumor fraction estimate using z-score statistics from sWGS data.

**Additional file 2: Fig. S2**

**Evaluation of the tumor fraction estimate determined by ichorCNA in cfDNA of healthy controls and cancer patients.**

(A) Tumor fraction estimated in healthy controls, cancer patients with or without mutations detected by cf-IMPACT. (B) Correlation of ichorCNA tumor fraction with genome-wide z-scores both computed from sWGS data. (C) Correlation of ichorCNA tumor fraction with median variant allele fraction calculated based on cf-IMPACT data of the same cfDNA sample.

**Additional file 2: Fig. S3. Distribution of genome-wide z-scores in patients with and without mutations detected by cfMSK-IPMACT.**

**A)** SWGS-estimated z-score distribution between plasma samples from healthy controls and cancer patients with or without mutations detected by MSK-IMPACT in the tumor. **B)** Correlation between sWGS-estimated z-scores and median variant allele fraction identified by cf-IMPACT as ND (Not detected, blue rectangles), <10% (green triangles) and ≥10% (red circles). Samples with and without matched tumor data available were shown separately in both panels.

**Additional file 2: Fig. S4. Comparison of proportion of tumor mutations detected in plasma with z-scores** ≥**5 and <5.**

Boxplots showing the proportion of mutations reported in the tumor (tumor mutations) detected in plasma with z-score less than 5, or equal to 5 or higher. Only patients with matched tumor data available were included in this analysis.

**Additional file 2: Fig. S5. Comparison of proportion of tumor mutations detected in plasma with tumor and plasma collected at different intervals**

Boxplots showing the proportion of mutations reported in the tumor (tumor mutations) detected in plasma in two groups: patients with plasma and tumor samples collected <180 days apart versus ≥180 days apart. Only patients with matched tumor data available were included in this analysis.

**Additional file 2: Fig. S6. Proportion of different categories of tumor mutations detected in the corresponding plasma sample.**

The proportion of tumor mutations detect in both tumor and plasma (Shared), tissue only (tissue private), or plasma only (plasma private) annotated in 5 groups: all mutations, hotspots, oncogenic, OncoKB level 1-4, and everything else.
